# Clot Retention: Our Experiences with a Simple New Technique of Evacuation with a Thoracic Catheter

**DOI:** 10.7759/cureus.4329

**Published:** 2019-03-27

**Authors:** Cemil Aydin, Aykut Bugra Senturk, Ali Akkoc, Ramazan Topaktas, Zeynep B Aydın, Musa Ekici

**Affiliations:** 1 Urology, Hitit University Erol Olcok Training and Research Hospital, Corum, TUR; 2 Urology, Alanya Alaaddin Keykubat University, Antalya, TUR; 3 Urology, Haydarpaşa Numune Training and Research Hospital, Istanbul, USA; 4 Radiology, Hitit University Erol Olcok Training and Research Hospital, Çorum, TUR

**Keywords:** clot retention, clot evacuation, thoracic catheter, hematuria

## Abstract

Introduction: Clot retention in the urinary bladder is a very common health problem in surgical and nonsurgical cases and clot retention treatment is quite costly.

Objectives: The aim of this retrospective study was to describe an alternative technique for removing tenacious and chronic clots by using a thoracic catheter technique.

Materials and methods: Between January 2011 and June 2018, a total of 27 patients of clot retention were treated under local anesthesia with the thoracic catheter technique.

Results: Twenty-seven patients with a mean age of 58 years (range 45-70) were included. The etiologies of bladder clots included surgical causes and nonsurgical causes. Of the surgical causes, the most common cause was post-transurethral resection of the prostate (TURP). The nonsurgical causes were upper tract bleeding, drug-induced bleeding, post-traumatic bleeding, and haematochyluria. It was found that the thoracic catheter technique was simple and easily adoptable, with no training required.

Conclusions: Clot retention in the urinary bladder is a very common problem in surgical and nonsurgical cases. Our technique is a simple, safe, fast, and effective option of clot removal from the urinary bladder and it doesn’t require any added cost.

## Introduction

Hematuria is an inevitable complication after transurethral resection of the prostate and carcinoma bladder. Hematuria with clot retention in the urinary bladder is a common health problem in surgical and nonsurgical cases worldwide. Bladder clots lead to urinary retention and present as an emergency [1]. It causes severe abdominal pain, hypertension, tachycardia, overdistended bladder and rarely may cause bladder rupture or perforation [1]. The standard management of clot retention is emergent clot evacuation using a Toomey syringe or Ellick’s evacuator. These procedures are highly effective, but sometimes these methods can fail and so some authors have described the use of alternate clot evacuation methods such as open suprapubic clot evacuation [1,2,3]. Multiple methods of clot evacuation have been described in the literature, and we report a method that is simple and easy to practice.

## Materials and methods

A total of 27 patients of clot retention were treated under local anesthesia with the thoracic catheter technique between January 2011 and June 2018. Approval was obtained from the local ethics committee, and written informed consent was obtained from all participants. Their mean age was 58 years (range 45-70 years). We retrospectively assessed the parameters of all the patients who underwent clot evacuation using the thoracic catheter technique.

Operative technique

**Figure 1:**
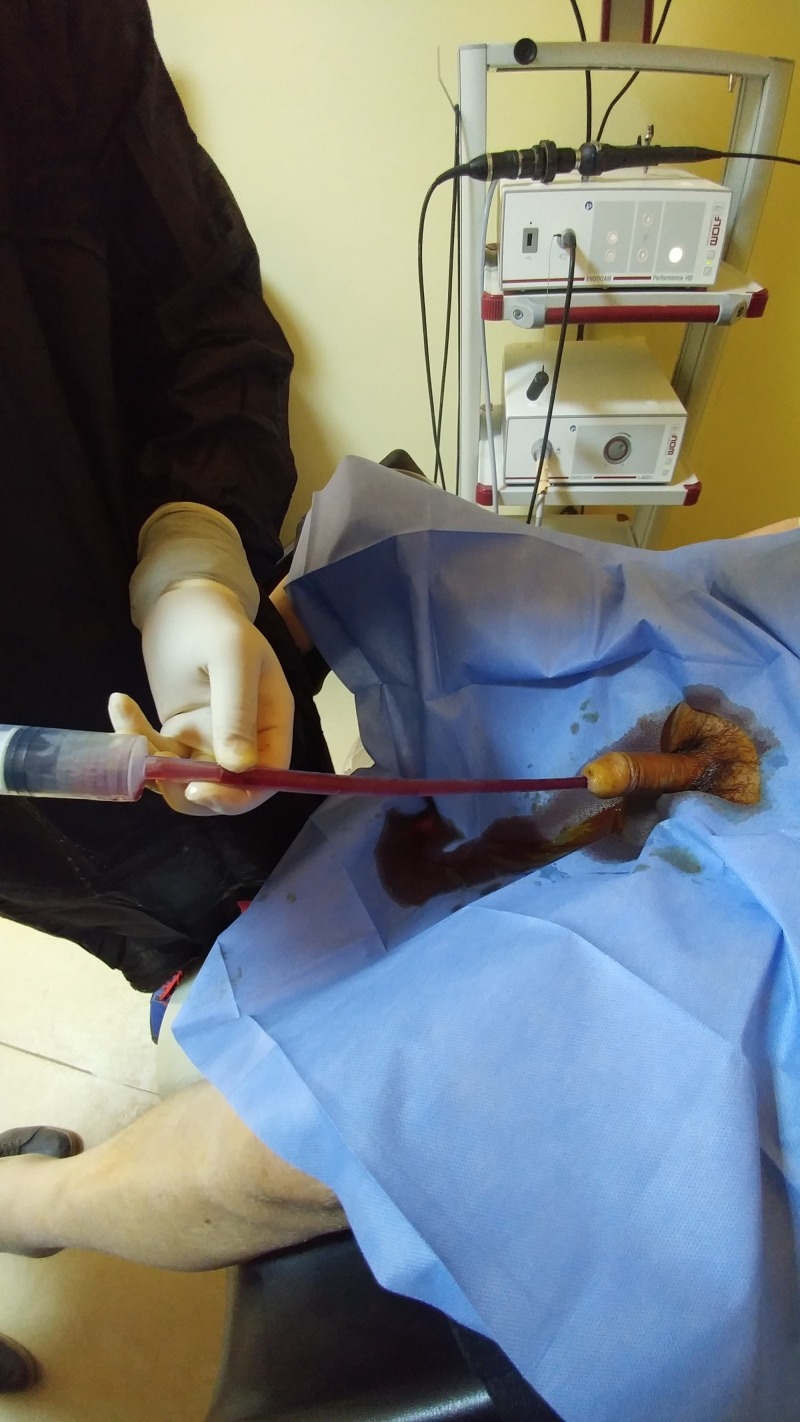
Operative photograph

The operative technique was as follows: the patients were kept in lithotomy position in an examination room or kept lying down on their own bed (Figure 1).

**Figure 2:**
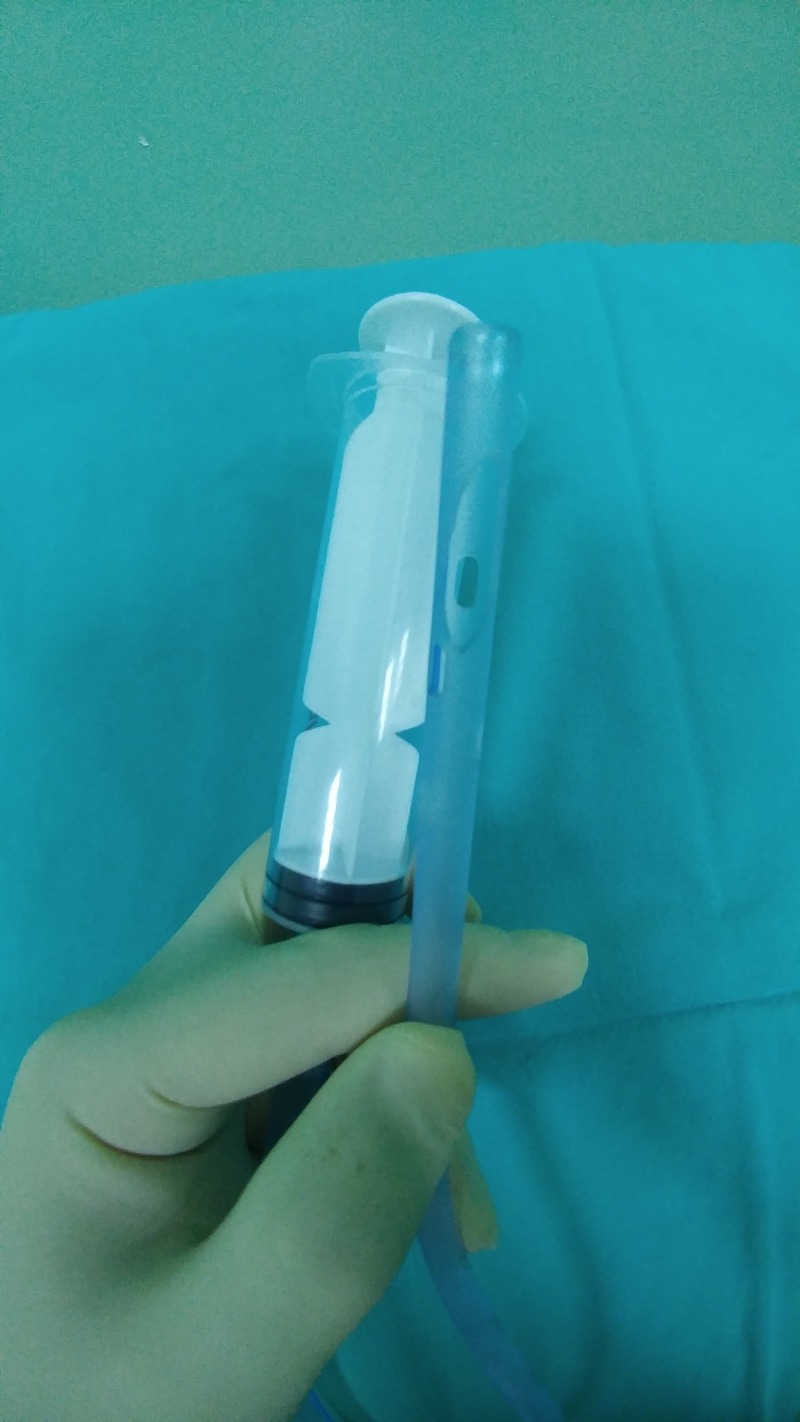
The tip of the thoracic catheter was cut into a semicircle

**Figure 3:**
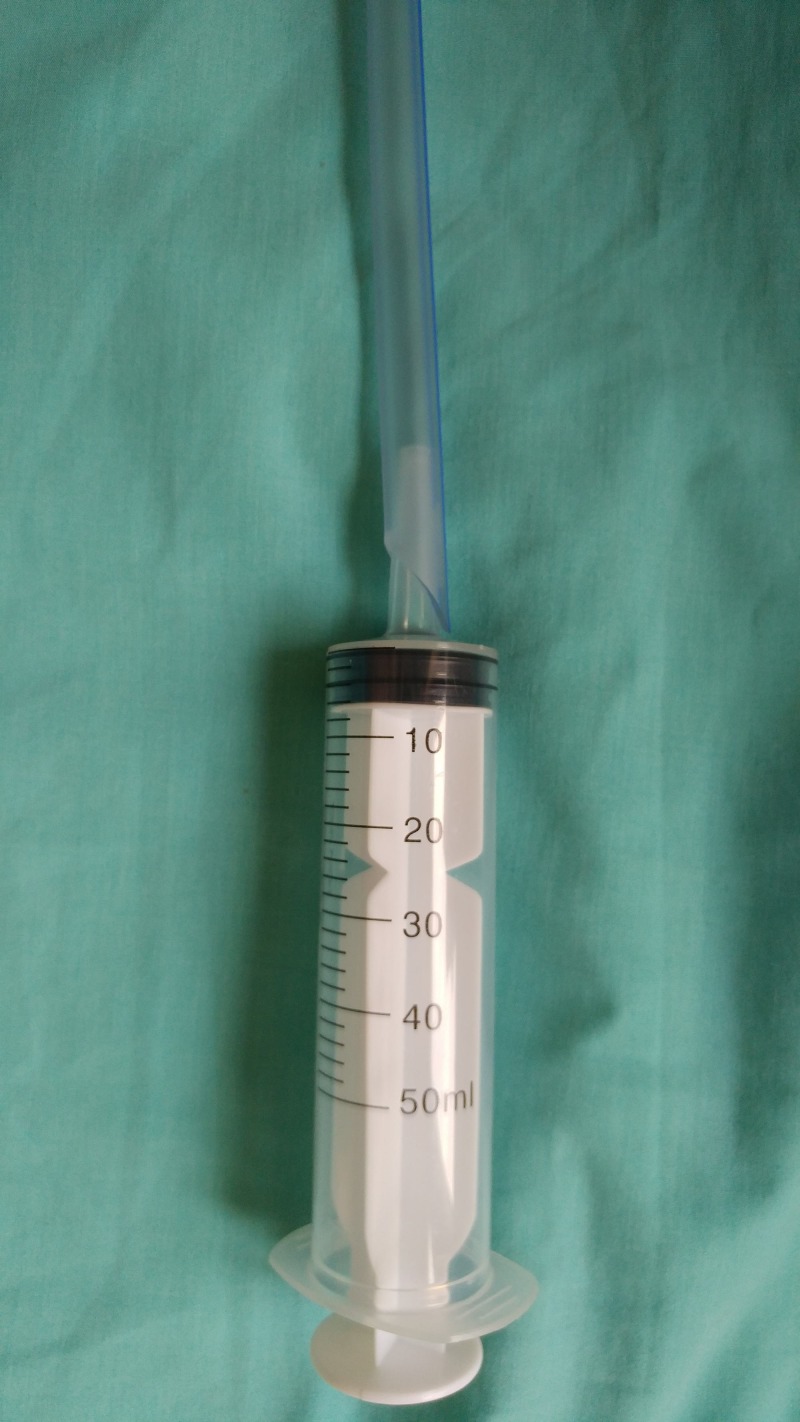
The thoracic catheter was connected to the plastic feeding syringe

The procedure was applied under local anesthesia by using 2% lidocaine gel, and a 22 or 24 French thoracic catheter was used. First, the tip side of the thoracic catheter was cut into a semicircle approximately 2 cm by scissors (Figure 2). The thoracic catheter was inserted into the bladder and then was connected to the plastic feeding syringe (Figures 1-3). For washing the bladder and evacuating the clot the suction was started by using sterile saline with a plastic feeding syringe. The clots were directly sucked. The tip of the thoracic catheter was moved sideways, up and down to further break the clots. We could see/ palpate the bladder being emptied. The thoracic catheter evacuated the clots without causing any trauma or injury to the bladder. At the end of the procedure, a three-way Foley catheter was inserted and bladder irrigation with normal saline was started. No patient required any additional clot extraction method to be used.

## Results

A total of 27 patients who were treated by this technic were included in this study. The etiologies of clot retention were post-transurethral resection of prostate bleeding 44.4% (12 men), bladder tumor 25.9% (one woman, total seven patients), post-transurethral resection of bladder tumor 14.8% (four patients), and others 14.8% (i.e., upper tract bleeding, drug-induced bleeding, post-traumatic bleeding, and haematochyluria -- two women, total four patients) (Table 1). The mean age of patients was 58 years (range 45-70 years). The mean duration of bladder clots was 18 hours (h) (range 10-28 h). The predicted clot size ranged from 100 milliliters (mL) more than 1 liter (L). The mean time for clot removal was 10 minutes (min) (range 5-25 min) (Table 2). All patients were clot-free at the end of this method. No complications such as urethra or bladder perforation were observed.

**Table 1:** Presentation of clot retention

TURP	TURBT	BLADDER TUMOR	OTHERS
12 (44.4%)	4 (14.8%)	7 (25.9%)	4 (14.8%)
TURP: Transurethral resection of the prostate, TURBT: Transurethral resection of a bladder tumor.			

**Table 2:** Patient characteristics and procedure details

GENDER (MALE/FEMALE)	24/3
Mean age, years (range)	58 (45-70)
Duration of clots, h (hours)	8 (10-28)
Predicted size of clots, L (liters)	0,1-1
Duration of procedure, minutes (range)	10 (5-25)

## Discussion

In most cases, removal of bladder clots is usually performed under general anesthesia by using a Toomey syringe or Ellick’s evacuator with cystoscopy. The Toomey syringe is better as the suction pressure generated is much higher compared to the Ellick’s evacuator. So using Ellick’s evacuator may be inadequate to suck old clots. Although the Toomey syringe and Ellick’s evacuator are safe, they can sometimes cause bladder perforation. We did not encounter such complications. Another method of removing tenacious clots is by using 0.15% or 0.3% hydrogen peroxide, streptokinase instillation in the bladder to lyse the clots [2,4], but we have no experience with this method. In the literature, Goel et al. performed mechanical suction to evacuate the clot [5]. In their method, either the 25-F cystoscope sheath or 26-F resectoscope sheath is left in the bladder and the rest of the instruments are removed; the clots are evacuated after directly connecting the suction tube with the sheath. They reported their technique as safe and successful and they did not encounter any complications. Inadequate control of bleeding during the intraoperative time can lead to postoperative bleeding. It may also arise from increased activity of the patient (i.e., forced straining with a bowel movement). So, laxatives may be recommended to reduce the strain on these patients. To the best of our knowledge we are the first to describe in the literature the use of thoracic catheter suction for the removal of tenacious clots with successful results. Clot evacuation using a thoracic catheter is safe, simple, and applicable. It doesn’t require any special skill to learn this method. We performed this technique with the patient under local anesthesia without the need for an operating room. The thoracic catheter technique is a blind procedure and it has certain disadvantages such as bladder injury or bladder perforation like any other conventional evacuation procedure (Ellick’s or Toomey). However, we did not encounter any serious complications. We did not include a price analysis in our study, but we think it is cheaper than other conventional methods. This study has certain limitations because there is no control group and it is a non-randomized study. Also, our study is a single center study including a relatively small number of patients.

## Conclusions

Clot retention, once it occurs, must be dealt with on an emergency basis. The thoracic catheter technique is a safe, fast, simple, and useful alternative technique of clot removal from the bladder. The advantages of this method are that it can be performed with local anesthesia and that it is feasible and cheaper than conventional techniques.
